# Ablation of hippocampal neurogenesis in mice impairs the response to stress during the dark cycle

**DOI:** 10.1038/ncomms9373

**Published:** 2015-09-29

**Authors:** Cheng-Yu Tsai, Ching-Yen Tsai, Sebastian J. Arnold, Guo-Jen Huang

**Affiliations:** 1 Department and Graduate Institute of Biomedical Sciences, College of Medicine, Chang Gung University, Tao-Yuan 33302, Taiwan; 2 Institute of Molecular Biology, Academia Sinica, Taipei 11529, Taiwan; 3 Renal Department, University Medical Centre, Centre for Clinical Research, Freiburg 79106, Germany; 4 Institute of Experimental and Clinical Pharmacology and Toxicology, Albert-Ludwigs-University, Freiburg 79102, Germany; 5 BIOSS Centre of Biological Signalling Studies, Albert-Ludwigs-University, Freiburg 79104, Germany; 6 Healthy Aging Research Center, Chang Gung University, Tao-Yuan 33902, Taiwan

## Abstract

The functional role of adult neurogenesis in the hippocampus remains the subject of intense speculation. One recent hypothesis is that adult-born neurons contribute to the endocrine and behavioural outputs of the stress response. Here we show a genetic model system to ablate neurogenesis by inducibly deleting *Tbr2* gene function specifically in the hippocampus and corroborate our findings in a radiation-based model of neurogenesis deprivation. We found that mice with ablation of new neurons in the dentate gyrus exhibit reduced anxiety during the dark cycle. After restraint stress, corticosterone levels in neurogenesis-deficient mice decreased more quickly than controls and were more sensitive to suppression by dexamethasone. Furthermore, glucocorticoid receptor target genes and neuronal activity markers showed reduced expression after stress in neurogenesis-deficient mice. These findings suggest that newborn neurons in the hippocampus are involved in sensing and eliciting an appropriate response to stress.

## Abstract (web summary)

Adult hippocampal neurogenesis is involved in the response to stress but whether it exerts a positive or negative effect remains unclear. Here the authors ablate hippocampal neurogenesis in mice and find that the effects on anxiety behaviour depend on the time of day, and that neurogenesis specifically impairs the response to stress during the dark cycle.

## Graphical abstract

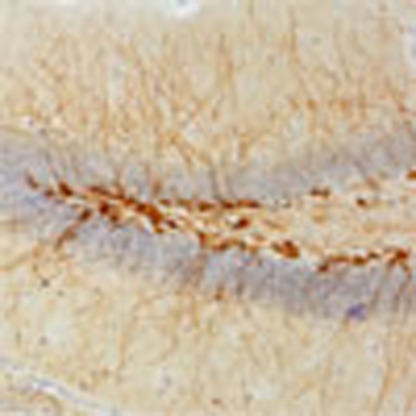

Evidence for adult neurogenesis was first published in the 1960s (ref. 1); however, scepticism about this claim persisted for nearly half a century because of experimental limitations. It is now well established that neurogenesis occurs throughout the lifespan of mammalian species, including humans^2^,^3^. However, discovering the functions of these newly born neurons remains an unmet challenge. The dentate gyrus (DG) in the hippocampus is one of the few places where adult neurogenesis takes place. The hippocampus is considered to play a part in learning and emotional regulation^4^. Thus, it is assumed that newly formed neurons in the adult hippocampus are related to learning and memory processes.

Psychiatric disorders, such as depression, are associated with stress, especially under chronic conditions^5^. However, the relationship between stress and neurogenesis is complex and reciprocal^6^. Numerous studies have shown that stress decreases adult neurogenesis in the hippocampus and the effect may depend on corticosterone^7^,^8^,^9^. Despite the large amount of studies showing that stress impairs neurogenesis, some studies found that stress had no effect, or actually even increased neurogenesis^10^,^11^. Intriguingly, chronic antidepressant treatment was reported to increase the level of neurogenesis in the adult rat hippocampus^12^. Therefore, researchers suggest that the clinical effects of antidepressants may be mediated through an increase in adult hippocampal neurogenesis^13^. Evidence from animal models supports this hypothesis. It has been reported that behavioural changes following treatment with antidepressants are lost in mice that have undergone hippocampal irradiation. This irradiation decreased 85% of cell proliferation in the hippocampus, suggesting that neurogenesis is required for antidepressants to evoke behavioural changes^14^. This hypothesis is attractive and may suggest an explanation for the delayed onset of the clinical effects of antidepressant drugs. It is noteworthy that others have cast doubts on this theory^15^,^16^. A recent study using transgenic mice that express herpes virus thymidine kinase (TK) under the regulation of the mouse Glial Fibrillary Acidic Protein (GFAP) promoter revealed that neurogenesis could buffer neuroendocrine responses to stress^17^. This study shed some light on the complexity of neurogenesis and the stress response. However, as these data were largely based on a single model, the GFAP-TK mouse, additional studies are needed to validate this conclusion. Despite a handful of studies having been published, the functions of these adult-born neurons are still largely unclear because of the lack of convincing animal models to approach this issue.

Understanding the underlying mechanisms and functions of newly formed neurons in the DG of the hippocampus may help devise strategies for clinical intervention in the treatment of stress-related disorders. Given the potential importance of this subject, it is necessary to further investigate the complicated relationship between neurogenesis and the stress response by using different animal models. In this study, we aimed to use an inducible genetic mouse model to deplete DG neurogenesis to study the importance of adult neurogenesis in response to stress. In addition, we used irradiation to impair adult neurogenesis in the hippocampus, thus providing an alternative approach. We revealed that mice without DG neurogenesis feature a reduced response to stress and less anxiety.

## Results

### Blockade of DG neurogenesis in Tbr2-inducible knockout mice

**Figure 1:**
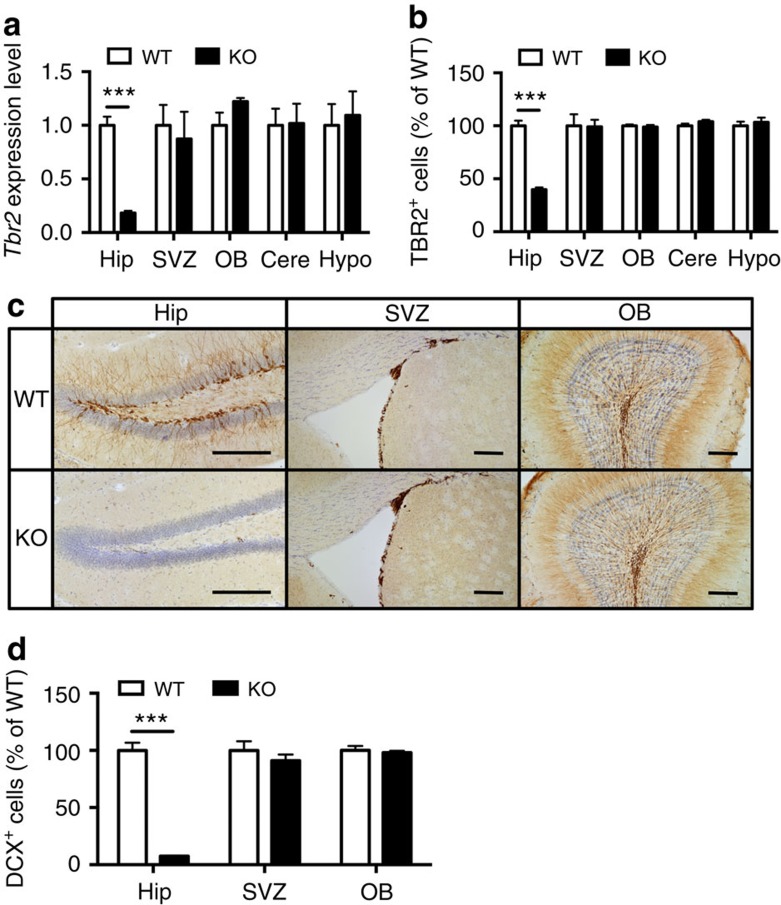
*Tbr2*-inducible KO mice show specific blockade of adult dentate gyrus neurogenesis. (**a**) Three weeks after the last tamoxifen injection, *Tbr2*-inducible KO mice (*n*=6) show an almost complete absence of *Tbr2* expression in the hippocampus (Hip), but not in the subventricular zone (SVZ), the olfactory bulb (OB), the cerebellum (Cere) and hypothalamus (Hypo). (**b**) *Tbr2*-inducible KO mice show a significant decrease in the number of TBR2^+^ cells in the hippocampus but not in other regions in the brain (*n*=5). (**c**) DCX immunostaining shows normal numbers of hippocampal immature neurons in WT mice while DCX^+^ cells are missing in KO mice. (**d**) After tamoxifen injection, *Tbr2*-inducible KO mice (*n*=5) exhibit a significant decrease in DCX^+^ cells in the dentate gyrus (*t*_8_=13.75, *P*<0.0001), but no difference in SVZ (*t*_8_=0.93, *P*=0.38) and OB (*t*_8_=0.46, *P*=0.66). Student's *t*-test, values represent mean±s.e.m. ***P<0.001. Scale bars, 200 μm.

Previous studies addressing the effects of adult DG neurogenesis were largely hampered by a lack of suitable model systems. Thus, we developed a genetic approach to ablate adult-born neurons specifically in the hippocampus by using tamoxifen-inducible *GLAST::CreER*^*T2*^; *Tbr2* floxed mice. *Tbr2* is a T-box transcription factor highly expressed in neuronal progenitors in the hippocampus^18^, and we have previously demonstrated that TBR2 is essential for adult neurogenesis^19^. Mice carrying the *GLAST::CreER*^*T2*^ allele express tamoxifen-inducible Cre recombinase in adult astrocytes as well as in radial glia cells^20^,^21^. As expected, 3 weeks after a final injection of tamoxifen to inducible *Tbr2* knockout (KO) mice, mRNA and protein levels of *Tbr2* were dramatically and specifically reduced in the hippocampus (Fig. 1a,b and Supplementary Fig. 1). To examine DG neurogenesis, we used doublecortin (DCX) as marker for immature neurons. The inducible *Tbr2* KO mice featured almost complete depletion of immature neurons in the DG, as evident by the decrease in DCX staining to less than 5% of control. No significant changes were observed in the other main brain regions that undergo adult neurogenesis such as the subventricular zone and olfactory bulb (Fig. 1c,d).

### Neurogenesis-deficient mice exhibit reduced anxiety

Our aim was to use this mouse model to study the relationship between adult neurogenesis and emotional behaviour. To investigate the long-term effect of adult neurogenesis depletion, we studied the *Tbr2* KO mice 10 weeks after their final tamoxifen injection. At this time, *Tbr2* KO mice, in comparison with wild type (WT), only showed 5% of DCX-positive cells (mean±s.e.m.; WT 2,259±145; KO 112±10, per DG) while body weight was unaltered (Supplementary Fig. 2a). Before we examined the anxiety behaviour, we measured 24-h locomotor activity. The result showed a trend towards increased activity of neurogenesis-deficient mice (*Tbr2* KO) during the dark cycle in comparison with WT mice (Supplementary Fig. 3a). We next used two paradigms to test anxiety-related behaviours: the elevated plus maze (EPM) and novelty-suppressed feeding (NSF). Both EPM and NSF results showed no significant difference between *Tbr2* KO and WT mice when tests were performed during the day (Supplementary Fig. 3c–f).

**Figure 2:**
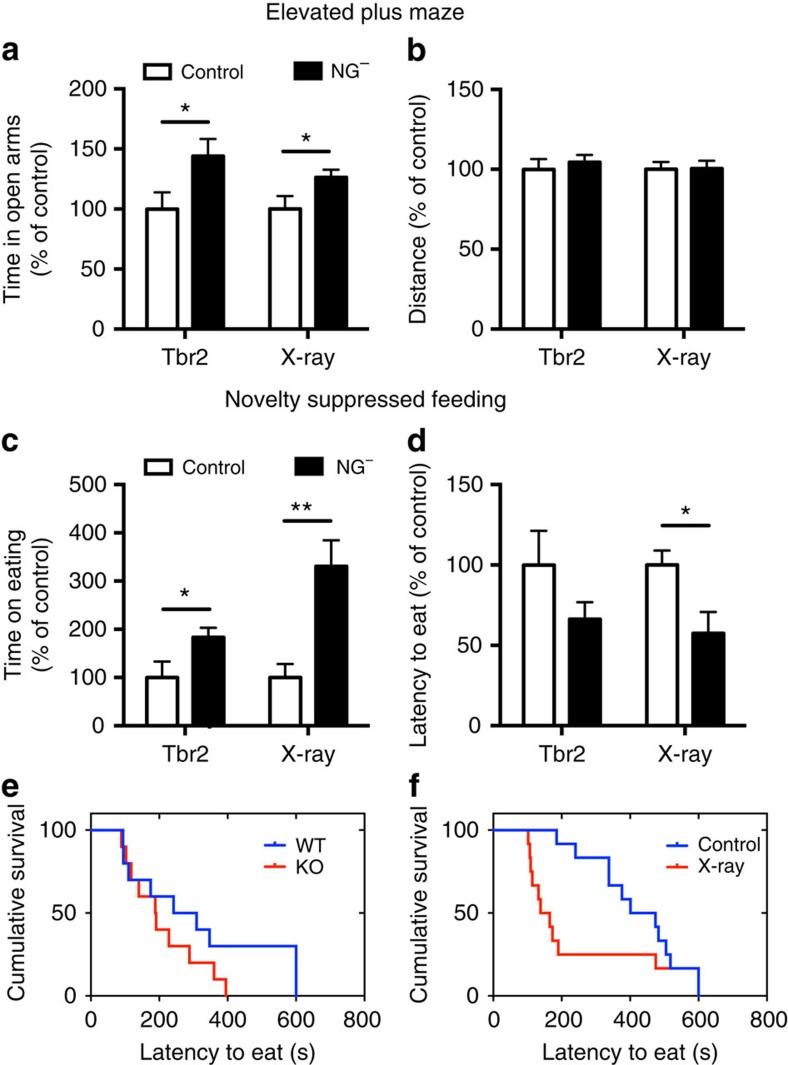
Mice lacking neurogenesis show reduced levels of anxiety. (**a**) Mice with neurogenesis ablation (NG^−^) spent more time in the open arms of the EPM test for both the *Tbr2* KO mouse model (*n*=10, *t*_18_=2.2, *P*=0.04) and irradiated mouse model (*n*=12 and 13, *t*_23_=2.06, *P*=0.05). (**b**) In EPM, no significant difference in travel distance for both *Tbr2* KO or irradiated mice was observed. (**c**) In the NSF test, both *Tbr2* KO and irradiated mice spent more time eating compared with the control during testing (Student's *t*-test, for *Tbr2* KO mice, *n*=10, *t*_18_=2.14, *P*=0.046; for irradiated mice, *n*=12, *t*_22_=3.78, *P*=0.001). (**d**) In the NSF test, there is a significant difference in latency to eat for irradiated mice (*n*=12, *t*_22_=2.2, *P*=0.038) but no difference in the *Tbr2* KO mouse model (Student's *t*-test, *n*=10, *t*_18_=1.42, *P*=0.17). (**e**,**f**) Time to begin eating as a survival plot in *Tbr2* KO mouse model (Gehan–Breslow–Wilcoxon test, *n*=10, *P*=0.407) and irradiated mouse model (Gehan–Breslow–Wilcoxon test, *n*=12, *P*=0.011). Values represent mean±s.e.m. *P<0.05, **P<0.01.

However, mice are nocturnal animals and our result from the 24-h locomotor activity recordings showed that KO mice exhibit increased activity at night. Consequently, we used an independent cohort of mice and repeated our measurements of anxiety behaviour during the dark cycle. In both tests, the *Tbr2* KO mice showed less anxiety-related behaviour. In the EPM*, Tbr2* KO mice spent more time in the anxiogenic open set of arms (Fig. 2a), with no difference in the total distance travelled compared with WT mice (Fig. 2b and Supplementary Fig. 4a; for absolute values see Supplementary Fig. 6). To assess whether the difference in time spent in the open arms was because of changed activity levels, we further analysed the total number of arm entries, and the results show no differences between the two groups (Supplementary Fig. 4c). This suggests that the difference in time spent in the open arms is not caused by changes in activity. Similarly, in the NSF test, *Tbr*2 KO mice spent more time eating food pellets in a new cage, which acts as an inducer of anxiety (Fig. 2c and Supplementary Fig. 6), despite no difference in latency to eat (Fig. 2d,e). When tested in their home cage, there was no difference in food consumption between WT and KO mice (Supplementary Fig. 5a). To further investigate whether neurogenesis-deficient mice exhibit differences in depression-like behaviours, mice were subjected to the forced swimming test and sucrose preference test. The results showed no difference between KO and WT mice (Supplementary Fig. 7). These experiments reveal that the blockade of adult DG neurogenesis influences anxiety behaviours, but not depression-like behaviours. To eliminate the possibility that the difference in anxiety between the WT and KO mice is driven by the expression of Cre, we tested the mice with oil injection instead of tamoxifen, which does not result in gene deletion of *Tbr2*. The result showed that there were no behavioural differences in activity, EPM and NSF (Supplementary Fig. 8). This indicates that the behavioural differences were caused by the inducible deletion of *Tbr2*, which results in the deficiency of neurogenesis in the DG.

To exclude the possibility that behavioural differences might result from the mode of neurogenic cell ablation, we repeated testing of anxiety levels using irradiated mice that similarly lack DG neurogenesis. DCX staining of irradiated mice demonstrated almost complete depletion of neurogenesis in the hippocampus to 1% of control levels, but only a moderate reduction to 75% in the subventricular zone, 3 weeks after two courses of 10-Gy irradiation (Supplementary Fig. 9). Mice were subjected to behavioural tests 10 weeks after irradiation. In agreement with the *Tbr2* KO model, the irradiated mice showed increased locomotor activity during the night (Supplementary Fig. 3b) and reduced anxiety in the EPM with no difference in activity measured by travel distance and total arm entries (Fig. 2a,b and Supplementary Fig. 4b,d; for absolute values see Supplementary Fig. 6). For NSF test, similar to *Tbr2* KO mice, irradiated mice also spent more time eating with less latency to approach food pellets (Fig. 2c,d,f;for absolute values see Supplementary Fig. 6). Previous irradiation studies that used either BALB/c or 129/SvEv animals showed no difference in anxiety tests between mice with and without neurogenesis when NSF or EPM experiments were conducted in the light cycle^14^,^22^,^23^. Our study used C57BL/6 mice and we detected no difference during the day, but neurogenesis-deficient mice showed significantly reduced anxiety-like behaviour during the night. Taken together, these two different models of neurogenesis ablation reveal that mice lacking adult neurogenesis behave less anxiously. This suggests that the presence of new DG neurons alters the behavioural response to mild stress.

### Neurogenesis-null mice show lower post-stress corticosterone

**Figure 3:**
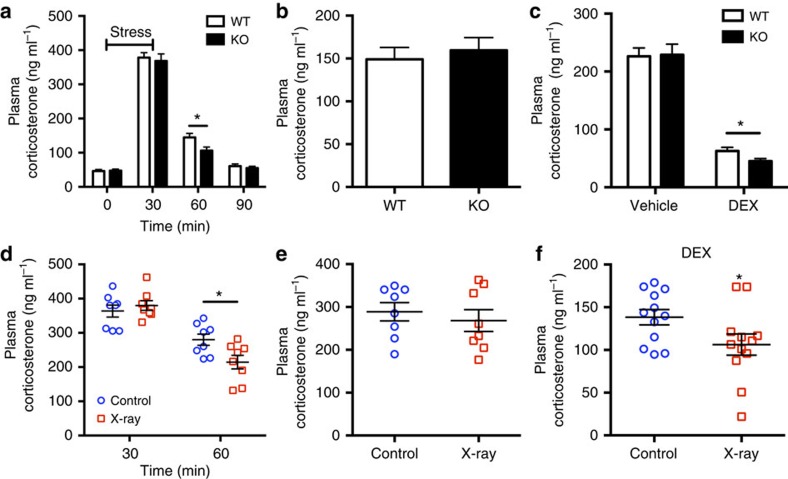
GR-mediated feedback inhibition of the HPA axis is enhanced in mice without neurogenesis. (**a**) Plasma corticosterone levels are reduced 30 min after the end of restraint stress in *Tbr2* KO mice (*n*=13 and 15, *t*_26_=2.4, *P*=0.024). (**b**) No difference in daytime plasma corticosterone 30 min after the end of stress for the *Tbr2* KO mouse model during the light cycle (*n*=8). (**c**) In the dexamethasone suppression test, 1 h after dexamethasone injection, *Tbr2* KO mice exhibited stronger suppression of corticosterone 10 min after onset of restraint stress (*n*=15 and 11, *t*_24_=2.2, *P*=0.038). (**d**) In irradiated mice, plasma corticosterone levels are reduced 30 min after the end of restraint stress in comparison with control mice (*n*=8, *t*_14_=2.55, *P*=0.023). (**e**) No difference in daytime plasma corticosterone level 30 min after the end of stress for the irradiated mouse model (*n*=8). (**f**) Irradiated mice show reduced levels of corticosterone in the dexamethasone suppression test (*n*=12, *t*_22_=2.11, *P*=0.047). Student's *t*-test, values represent mean±s.e.m. *P<0.05.

The hippocampus is a key region for the feedback regulation of the hypothalamic–pituitary–adrenal (HPA) axis in response to stress^24^. Previous studies suggested that adult neurogenesis influences stress-induced alterations in HPA axis activity^17^. To further investigate the effects of adult neurogenesis on the HPA axis response to stress, we subjected mice to restraint stress and measured plasma corticosterone levels at different time points in the dark cycle. As expected, corticosterone levels increased dramatically after stress and recovered after 90 min. While there was no difference in the corticosterone levels between WT and KO mice before stress and after 30 min, significant differences were observed during the recovery phase after 60 min (Fig. 3a). Of note, these results differ from a previous report^17^. Thus, for comparisons across studies, we used another cohort of mice and repeated the stress experiment during the day cycle. We collected the blood sample at the 60-min time point, and, interestingly, unlike corticosterone levels during the night, no difference between WT and KO mice was detected during the day (Fig. 3b). We next tested feedback inhibition of the HPA axis by using a dexamethasone suppression test in the dark cycle. Dexamethasone is a potent agonist for the glucocorticoid receptor (GR). One hour before restraint stress, we pretreated mice with dexamethasone to suppress corticosterone. Dexamethasone effectively suppressed plasma corticosterone levels after a 10-min restraint stress (Fig. 3c). However, the plasma corticosterone levels in dexamethasone-injected KO mice were significantly reduced in comparison with dexamethasone-injected WT mice after stress (Fig. 3c).

To corroborate our findings of enhanced recovery after stress and the effect of dexamethasone in *Tbr2* KO mice, we ablated adult neurogenesis using the irradiation model to confirm the effects of neurogenic ablation. Strikingly, we observed a very similar effect to that seen with *Tbr2* KO animals: compared with controls, the irradiated mice had lower levels of plasma corticosterone 60 min after stress in the dark cycle; however, no difference was detected in the day cycle (Fig. 3d,e). In addition, dexamethasone-injected irradiated mice also had lower levels of corticosterone after stress in the dark cycle (Fig. 3f). These results suggest that the increase in sensitivity to GR-mediated stress suppression observed in neurogenesis-deficient mice was not related to the method used for the depletion of newborn neurons. Moreover, these results demonstrate that the GR-mediated feedback inhibition of the HPA axis is regulated by newly born neurons during the night, but not during the day.

### Mice with neurogenesis exhibit greater responses to stress

**Figure 4:**
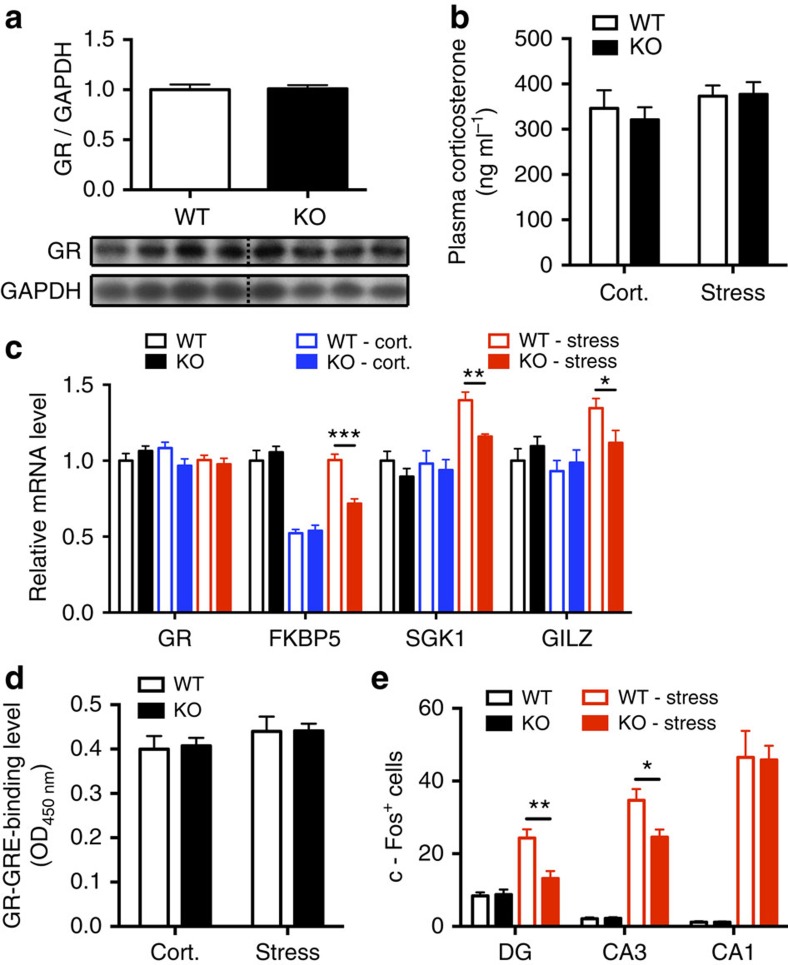
Mice with neurogenesis exhibit a greater response to stress in GR target gene expression and hippocampal neuronal activity assays. (**a**) Western blot analysis of the GR protein level in the hippocampus does not show differences between KO and WT (*n*=7). (**b**) Mice were injected with 0.25 mg kg^−1^ corticosterone and corticosterone levels were measured 30 min after injection. No significant differences between WT and KO mice were observed (*n*=8–10). (**c**) The hippocampal gene expression of GR (*Nr3c1*) and GR target genes 30 min after stress or corticosterone injection were analysed by qRT–PCR. There were no differences in GR expression among all the groups. However, the gene expression of GR target genes is increased in WT mice compared with KO mice after stress (*n*=10 and 8, FKBP5, *t*_16_=5.61, *P*<0.0001; SGK1, *t*_16_=3.82, *P*=0.001; GILZ, *t*_16_=2.26, *P*=0.038), indicating that stress resulted in higher expression of GR target genes in mice that exhibit hippocampal neurogenesis. (**d**) GR-GRE-binding assay revealed no differences between WT and KO mice 30 min after corticosterone injection or restraint stress stimulation (*n*=8–9). (**e**) Ninety minutes after stress started, mice with neurogenesis depletion showed less c-Fos^+^ cells in the DG (*n*=8 and 7, *t*_13_=3.47, *P*=0.004) and CA3 (*t*_13_=2.64, *P*=0.02), but no difference in the CA1(*t*_13_=0.08, *P*=0.93). Student's *t*-test, values represent mean±s.e.m. *P<0.05, **P<0.01, ***P<0.001.

One possible explanation for the increased feedback regulation of the HPA axis following ablation of neurogenesis might be an increased expression, or increased activity of hippocampal GR. To test this possibility, we first measured hippocampal GR protein levels using western blot analysis. WT and KO mice showed similar GR protein levels in the hippocampus (Fig. 4a and Supplementary Fig. 12) and in the DG (Supplementary Fig. 10a and Supplementary Fig. 12) during the night. Next, we assessed expression of target genes of the activated GR nuclear complex (*Fkbp5*, *Sgk1* and *Gilz*), 30 min after corticosterone injection or stress. To elicit the direct effect of corticosterone, we injected corticosterone subcutaneously to reach levels similar to those occurring during stress. Accordingly, levels of plasma corticosterone 30 min after injections (0.25 mg kg^−1^) were similar to the levels measured after 30 min of restraint stress (Fig. 4b). No significant differences in the expression of GR target genes could be found after corticosterone injections between WT and KO. However, after restraint stress, the expression of all GR target genes examined was significantly higher in WT mice compared with KO mice (Fig. 4c). To assess whether those genes are specifically affected by GR actions, all the mice were pretreated with the GR antagonist RU486 (40 mg kg^−1^) 1 h before they were subjected to stress or corticosterone injection. The results showed that changes to these GR target genes were negated (Supplementary Fig. 10b). This suggests that corticosterone is required to elicit the effect of stress on GR target gene expression but is not the only factor that regulates their expression. In addition, we assessed GR activity in the hippocampus by measuring nuclear GR binding to synthetic oligonucleotides containing the glucocorticoid response elements (GRE). However, we found no significant differences in the expression or the activation of the GR between WT and KO 30 min after corticosterone injection or stress (Fig. 4c,d).

Our data indicate that neurogenesis-ablated mice behave less anxiously and have lower expression of stress-induced GR target genes. To better understand the neuronal activity in WT and KO hippocampi in response to stress, we assayed stress-induced neuronal activation by measuring the number of c-Fos-positive cells in different regions of the hippocampus. The expression of c-Fos is commonly used as a marker for neuronal activity following a stimulus^25^. We stained the hippocampus for c-Fos under basal conditions or 90 min after stress, at the time of peak expression after stimulation^26^. In accordance to our previous results, the numbers of c-Fos-positive cells in the DG and CA3 after stress stimulation were significantly increased in WT, while no significant difference in CA1 was observed (Fig. 4e and Supplementary Fig. 11). This suggests that mice deficient in hippocampal neurogenesis show lower hippocampal activity in response to stress.

## Discussion

Our results suggest that adult neurogenesis in the hippocampus is involved in sensing or responding to stress. Two independent mouse models for the ablation of DG neurogenesis showed reduced anxiety and a faster plasma corticosterone recovery following stress. Mice lacking neurogenesis also exhibit reduced expression of GR target genes and a decline in neuronal activity after stress. A recent study by Groves *et al*.^27^ demonstrated that GFAP-TK rats exhibited reduced anxiety in two NSF tests and a black/white alley emergence test. Despite differences in the properties of adult-born neurons between these two species^28^, our results are consistent with behavioural data from GFAP-TK rats. Another study by Lagace *et al*.^11^ also demonstrated that ablation of adult neurogenesis via X-ray irradiation decreased the proportion of mice susceptible to stress-induced avoidance, which involves memory processing of a previous experience of social defeat. The novelty of our study includes the introduction of a new model to study neurogenesis. In addition, our results show that the time of day is critical in influencing the effects of DG neurogenesis on anxiety. Thus, our study reveals one of the sources for the heterogeneity of results between studies on adult neurogenesis.

A previous study by Snyder *et al*.^17^ suggests that DG neurogenesis buffers the stress response and is protective against depression-like behaviour. In contrast to our study, they showed a higher level of plasma corticosterone in neurogenesis-deficient mice compared with control mice after stress, and impaired dexamethasone suppression of the restraint-induced rise in corticosterone. However, the two studies had several methodological differences that may account for the different outcomes. In the study by Snyder *et al*., mice were housed singly in a new cage after stress. This may also introduce further stress, explaining increased plasma corticosterone level 30 min following the experimentally induced stress. In our study, mice were returned to their own home cage after stress. Also different from Snyder *et al*. we conducted stress experiments and behavioural assays in the dark cycle (8:00–10:00 p.m.). Since mice are nocturnal animals, testing them at night is more reflective of their aroused state. Experiments during the daytime may introduce additional confounding effects. However, discrepancies between studies during the day or at night may point towards additional unknown mechanisms. In our study, unlike the results of experiments conducted during the night, we detected no difference in plasma corticosterone 60 min after stress in both animal models tested during the day. This is consistent with a previous study by Surget *et al*.^29^, which found no difference in dexamethasone suppression between irradiated and non-irradiated mice during the daytime. Together with our behavioural data that showed that differences between neurogenesis-proficient and -deficient animals were only evident during the night, these results raise the possibility that newly formed neurons in the DG mediate the HPA axis and anxiety responses during the night when mice are awake. Intriguingly, the circadian clock also regulates neurogenesis by controlling the entry and exit of neuronal progenitor cells into the cell cycle, and higher levels of proliferation occur at night^30^, suggesting that circadian rhythmicity not only regulates neurogenesis but may also mediate the functions of newly generated neurons on behaviour and physiology.

One of the limitations of this study was that a single strain of inbred mice was used in experiments. It is well accepted that gene × gene interactions are crucial in influencing physiology and behaviour. However, we observed consistent results in two different models tested under more physiological, nocturnal conditions; our results provide evidence that mice with normal adult DG neurogenesis respond to stress more strongly in comparison with mice that lack DG neurogenesis. One possible explanation may lie in an enhanced susceptibility to the activation of newborn neurons by stimulation as previously shown^31^. A functioning stress response is vital for animal survival. Together, this study provides evidence that adult DG neurogenesis is required to mount an appropriate reaction to stress rather than simply decreasing the response to stress.

## Methods

### Animals

Four to six mice per cage were housed in a 12-h:12-h light/dark cycle (07:00 a.m. to 07:00 p.m.) at 22 °C and 60% humidity. Animals had access to food and water *ad libitum*. *Tbr2*^*CA/CA*^ mice had been backcrossed to C57BL/6 for more than six generations. *GLAST::Cre*^*T2*^ mice were generated from C57BL/6 ES cells^20^. *Tbr2*^*CA/CA*^*;GLAST::CreER*^*T2*^ and *Tbr2*^*CA/CA*^ mice used in this study were littermates generated by crossing *Tbr2*^*CA/CA*^;*GLAST::CreER*^*T2*^ mice with homozygous *Tbr2*^*CA/CA*^. All protocols used in this study were reviewed and approved by the Institutional Animal Care and Use Committee at the Chang Gung University, Taiwan (Permit Number: CGU13-067).

### Tamoxifen injection

*Tbr2*^*CA/CA*^*;GLAST::CreER*^*T2*^ and control *Tbr2*^*CA/CA*^ mice (male, littermates, 7-week old) were subjected to daily intraperitoneal injection of tamoxifen (150 mg kg^−1^) for 4 days, and injections were repeated after 1 week for another 4 days.

### Irradiation

C57BL/6 mice (male, 7-week old) were irradiated under anaesthesia by a mixture of Zotetil and rompun and were restrained by adhesive tape during irradiation. In this study, we used an UNIQUE linear accelerator (Varian medical systems). Mice were irradiated with 10 Gy in the hippocampal region from the linear accelerator on two consecutive days.

### Immunohistochemistry

All sections for DCX, TBR2 and c-Fos staining were cut at a thickness of 40 μm on a sliding microtome. Sections were mounted on SuperFrost slides and dried overnight. Subsequently, slides were incubated in 0.01 mol l^−1^ citric buffer for 40 min at 90 °C, 3% H_2_O_2_ for 10 min, rinsed in PBS and incubated overnight at room temperature in DCX antibody (1:400, Santa Cruz), c-Fos antibody (1:1,000, Santa Cruz) or TBR2 antibody (1:1,000, Abcam). Next day, a standard IgG ABC kit (Vector Lab) procedure was used, and the slides were incubated for 5–10 min with a Sigma DAB tablet. Sections were then counterstained with cresyl violet and mounted with mounting medium (DPX).

### Corticosterone assay

Blood samples were collected between 8:00 and 10:00 a.m. or 8:00 and 10:00 p.m. using facial vein puncture. Plasma was separated from whole blood by centrifugation and was stored at −80 °C until used. To analyse, plasma was diluted 1:40 in buffer and was measured using the Corticosterone EIA kit (Enzo Life Sciences) according to the manufacturer's instructions.

### Western blot

Tissue from the hippocampus was homogenized in 1% SDS solution and was boiled for 5 min. Protein concentration was quantified using the Bradford protein assay. Equal amounts of protein (30 μg) were separated with 10% SDS–polyacrylamide gel electrophoresis and were transferred on polyvinylidene difluoride membrane. Membranes were incubated with 5% non-fat milk powder for 1 h at room temperature and overnight at 4 °C with primary antibodies against GR (1:200, Santa Cruz Biotechnology) or GAPDH (1:5,000, Cell Signaling Technology). After washes, membranes were incubated with horseradish peroxidase-conjugated secondary antibody (1:1,000, Pierce Biotechnology) for 1 h at room temperature. Blots were developed using the enhanced chemiluminescence substrate kit (Amersham Biosciences) and exposed to an X-ray film (Kodak). The GR protein intensity signals were quantified using the Image-J software and were normalized against the GAPDH control.

### GR-GRE-binding assay

A TransAM GR kit (Active Motif) was used to measure nuclear GR binding to GRE consensus sequences. Thirty minutes after corticosterone injection or restraint stress, the hippocampus was dissected. Brain tissue was homogenized following the protocol from the Nuclear Extract Kit (Active Motif). The protein concentrations of nuclear extracts were determined using the Bradford protein assay, and 5 μg of nuclear extract protein was used. Absorbance readings for TransAM GR assays were taken with the absorbance wavelength set at 450 nm with a reference wavelength of 655 nm.

### Elevated plus maze

The apparatus used for the EPM test consisted of two open arms (30 × 5 cm) and two enclosed arms of the same size, with 15-cm-high transparent walls. The maze was elevated 38 cm above the floor. Mice were placed in the EPM for 5 min with dim light. Behaviour was tracked using the Ethovision software and the time spent in the open arm was measured.

### Novelty-suppressed feeding

Food pellets were removed from the home cage for 24 h before testing. For testing, mice were placed in a corner of the white open field apparatus (50 cm height, 60 cm diameter) with bright light and a single food pellet placed on white filter paper (9 cm diameter). The floor was covered with 1 cm of wooden bedding. The latency to eat and time spent on eating in the 10-min test period was recorded manually using the stopwatch. Food consumption in the home cage was measured for 10 min after 24 h fasting.

### Twenty-four-hour locomotor activity

To test the basal activity, two mice in the same experimental group were placed together in an activity cage with adequate food and water. Locomotor activity was then recorded after a 10-h habituation period using the Ethovision software. During the dark cycle, the tests were carried out under dim red light.

### Forced swimming

The apparatus consisted of two plastic cylinders (50 cm height × 20 cm diameter). The cylinders were filled with water, up to a height of 15 cm. Mice were placed into the cylinders, and their behaviour was recorded over a 5-min test period. Data acquisition and analysis were performed automatically using the EthoVision software.

### Sucrose preference

The method was applied as previously published^17^ for a 10-min measurement. Briefly, mice were individually housed and offered two water bottles containing water or 1% sucrose. After habituation for 3 days, both bottles were removed at 12:00 p.m. and the sucrose preference test was started at 7:00 p.m. for 10 min after putting back the two bottles. The weight changes of the two bottles during 10 min were measured and sucrose preference was expressed as (Δweightsucrose)/(Δweightsucrose+Δweightwater) × 100.

### mRNA quantification

Hippocampus tissues were collected and mRNA was extracted using a RNeasy Lipid Tissue Kit (Qiagen). cDNA was synthesized using SuperScript III reverse transcriptase (Invitrogen). Expression was measured using quantitative PCR using the following primers:

*Gapdh* (F: 5′-CTAGAGAGCTGACAGTGGGTAT-3′, R: 5′-AGACGACCAATGCGTCCAAA-3′),

*Tbr2* (F: 5′-TCCTAACACTGGCTCCCACT-3′, R: 5′-TTGTGCAGAGACTGCAACACT-3′),

GR (*Nr3c1*) (F: 5′-ACGCCGACTTGTTTATCTGG-3′, R: 5′-GAAAAGGACGCCAGACTCC-3′),

*Fkbp5* (F: 5′-AGCACACATCCCGTGTTCTA-3′, R: 5′-TGCTGGGTTCTCTCCATTGT-3′),

*Sgk1* (F: 5′-CTGCTCGAAGCACCCTTACC-3′, R: 5′-TCCTGAGGATGGGACATTTTCA-3′),

*Gilz* (F: 5′-AACACCGAAATGTATCAGACCC-3′, R: 5′-GTTTAACGGAAACCAAATCCCCT-3′),

Gene expression levels were calculated by the ^ΔΔ^*C*_t_ method and normalized against a *Gapdh* control.

## Additional information

**How to cite this article:** Tsai, C.-Y. *et al*. Ablation of hippocampal neurogenesis in mice impairs the response to stress during the dark cycle. *Nat. Commun*. 6:8373 doi: 10.1038/ncomms9373 (2015).

## Supplementary Material

Supplementary Information Supplementary Figures 1-12
