# Supplementary material for: Ablation of hippocampal neurogenesis in mice impairs the response to stress during the dark cycle
Source: Nat Commun. 2015 Sep 29;6:8373. doi: 10.1038/ncomms9373 (PMC4598562; doi:10.1038/ncomms9373)
Supplement: Supplementary Information — Supplementary Figures 1-12 [file ncomms9373-s1.pdf]

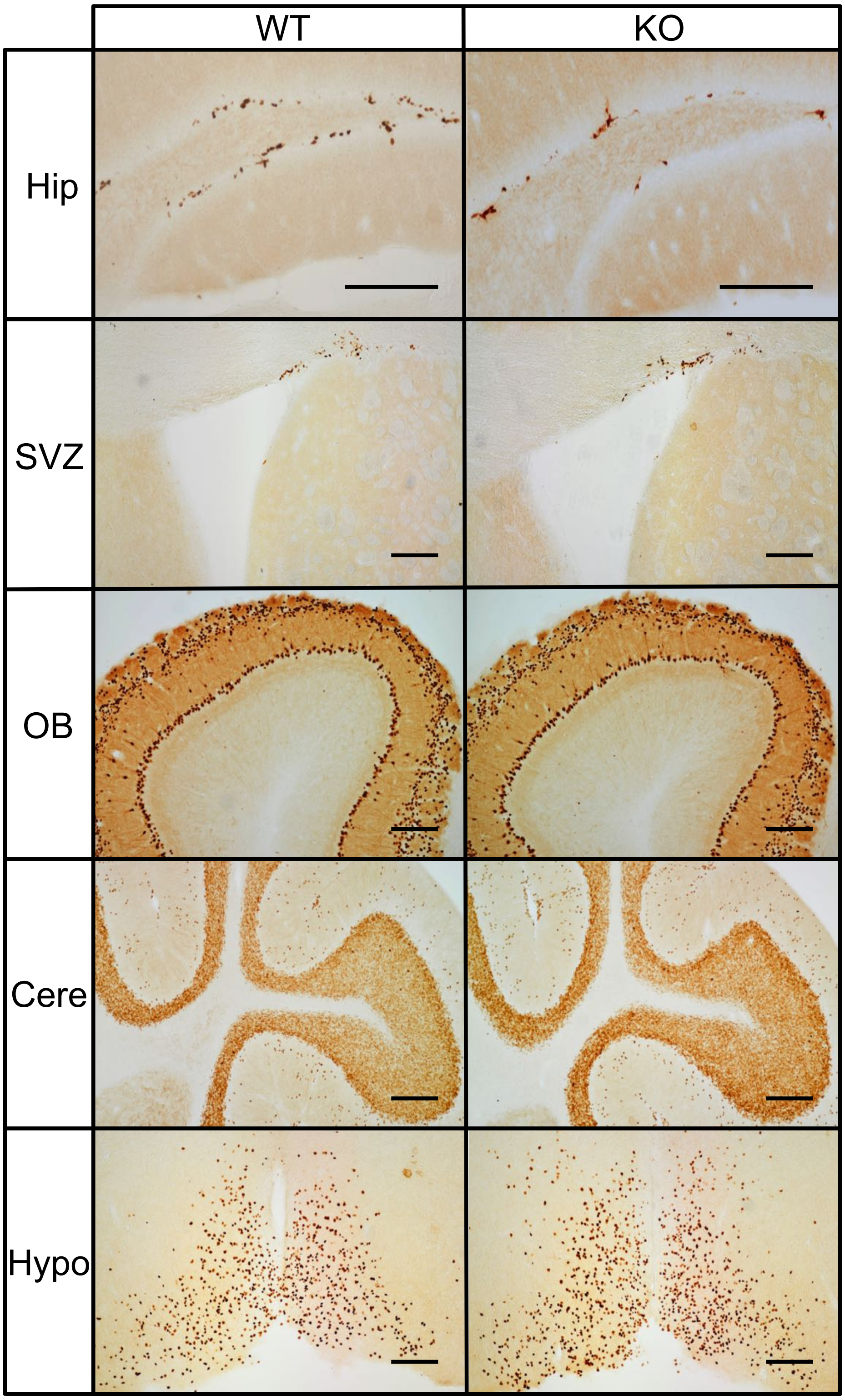

Supplementary Fig. 1: TBR2^{+} cells in different brain regions.

Three weeks after the last tamoxifen injection, TBR2 immunostaining images reveal a large reduction of TBR2^{+} cells in the dentate gyrus but not in the subventricular zone, olfactory bulb, cerebellum, and hypothalamus. Scale bars = 200 μm.

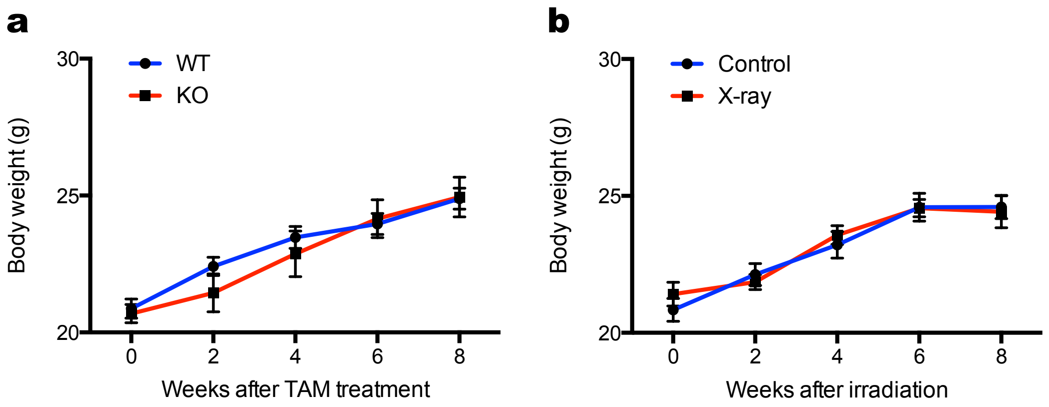

Supplementary Fig. 2: Body weight after TAM treatment or irradiation.
(a) No difference in body weight between *Tbr2* KO and WT mice (n = 9 and 11). (b) No difference in body weight between control and irradiated mice (n = 11 and 9).

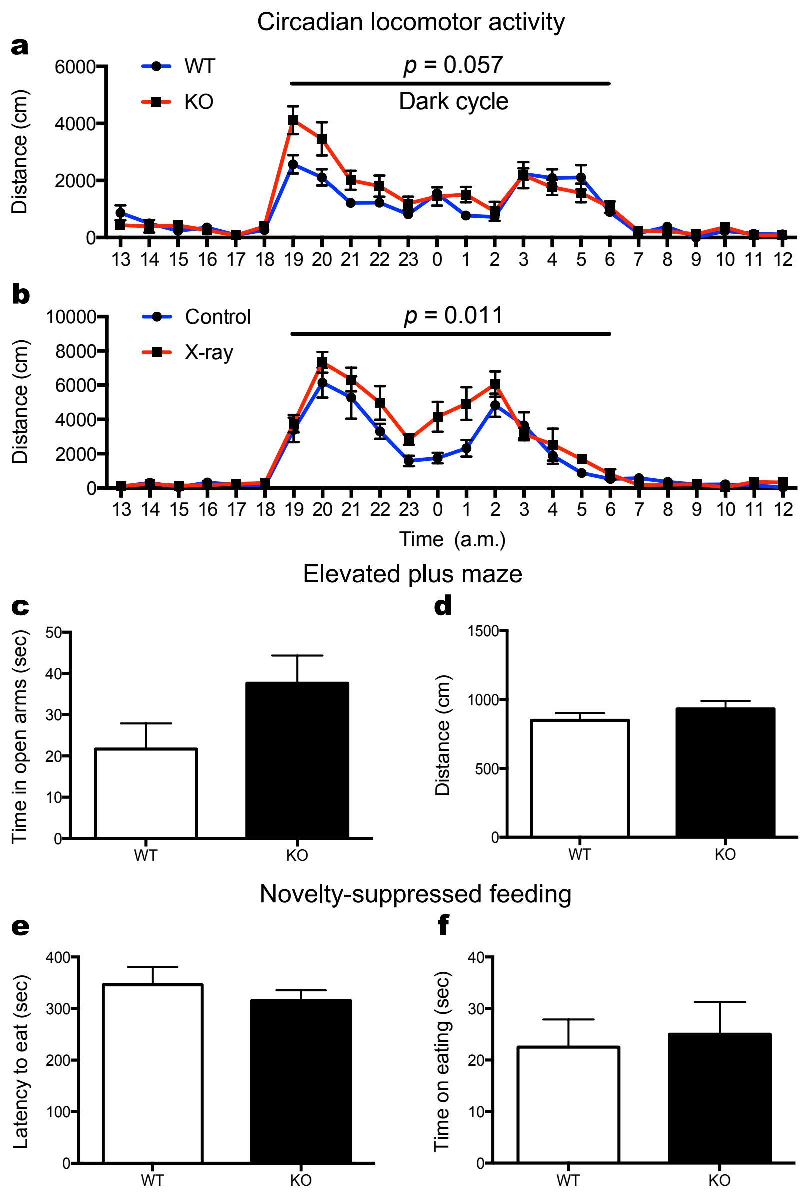

Supplementary Fig. 3: Locomotor activity and anxiety tests.
(a) 10 weeks after tamoxifen injection to deplete hippocampal neurogenesis, *Tbr2* KO mice have a trend towards a higher activity level compared to WT mice in the 24-hour locomotor activity test (n = 8, Repeated Measures ANOVA; effect of genotype, $F_{1,154}$ = 4.28, p = 0.057) in the dark cycle. (b) 10 weeks after X-irradiation on the hippocampus to block neurogenesis, irradiated mice show increased activity in the locomotor activity box in the dark cycle compared to sham mice (n = 8, Repeated Measures ANOVA; effect of X-ray, $F_{1,154}$ = 8.58, p = 0.01). (c, d) In the EPM test during the day, there was no differences in time spent in the open arms or the distance travelled between KO and WT mice (n = 10). (e, f) In the NSF test during the day, there was no differences in latency to eat or time spent on eating between WT and KO mice (n = 10).

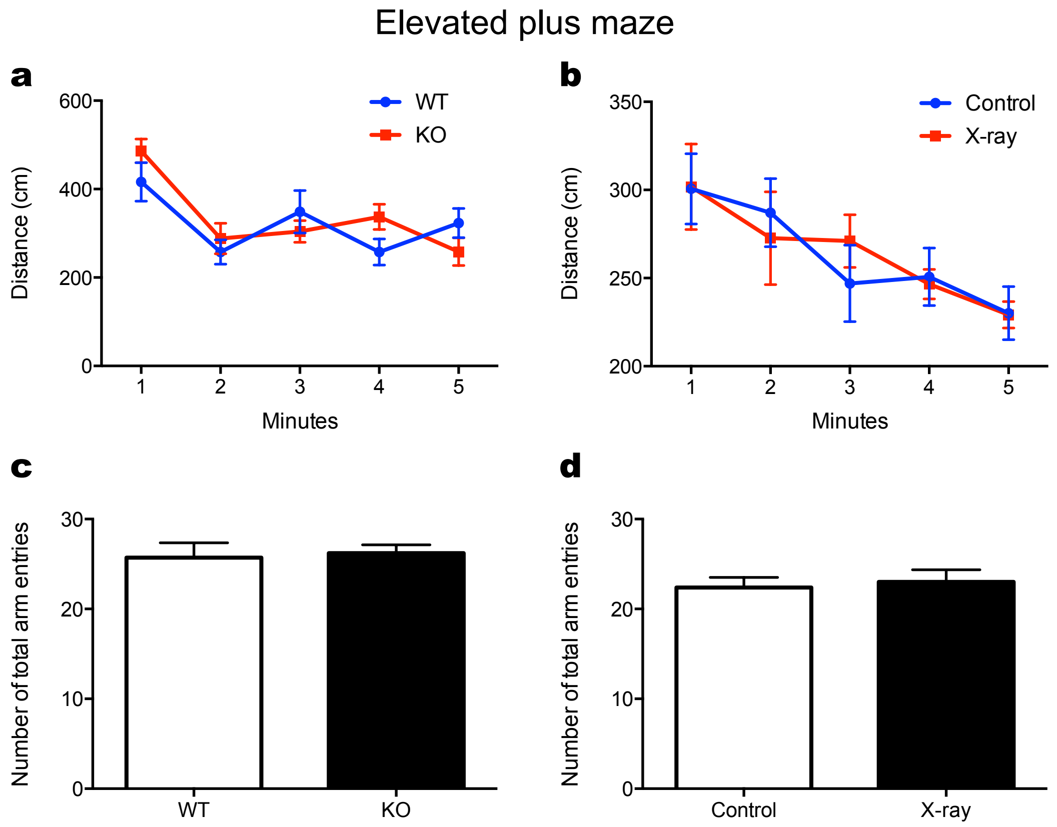

Supplementary Fig. 4: Activity in elevated plus maze.

No differences in total distance travelled (a, b) in both *Tbr2* mouse model (KO vs WT, n = 10) and irradiated mouse model (Control vs X-ray, n = 12), nor the total number of arm entries in both groups (c, d).

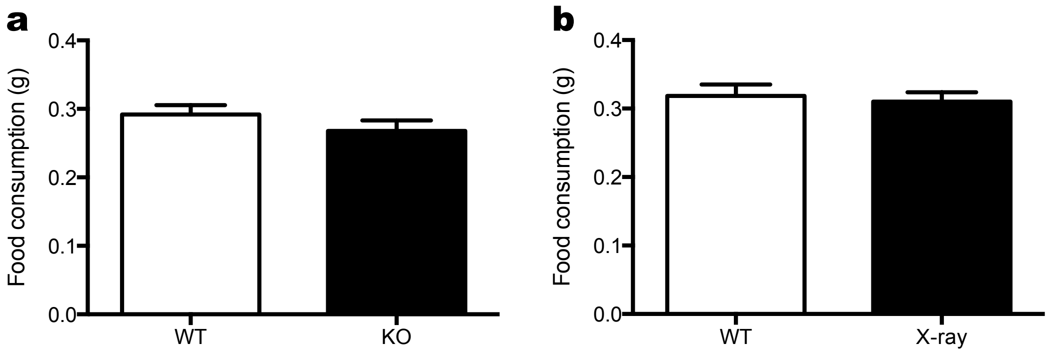

Supplementary Fig. 5: Home cage food consumption.
There are no significant differences in food consumption within 10 min after 24 h food deprivation for *Tbr2* KO animal model (n = 10) (**a**) and irradiation animal model (n = 12) (**b**) in their home cage.

	EPM		NSF	
	Time in open arm (sec)	Distance (cm)	Time on eating (sec)	Latency (sec)
WT	19.7 ± 2.7	1603 ± 101	53.5 ± 17.8	317.2 ± 67.4
KO	28.4 ± 2.8	1675 ± 73	98 ± 10.8	210 ± 33.9
Control	43.6 ± 4.7	1316 ± 61	44.5 ± 12.6	421.5 ± 38.1
X-ray	55.1 ± 2.8	1322 ± 64	147.1 ± 24	242.1 ± 56.3

Supplementary Fig. 6: Absolute values of anxiety behaviors.
Absolute values of elevated plus maze and novelty suppressed feeding tests for Tbr2 mice (WT vs KO) and irradiated mice (Control vs X-ray). Values represent mean ± SEM.

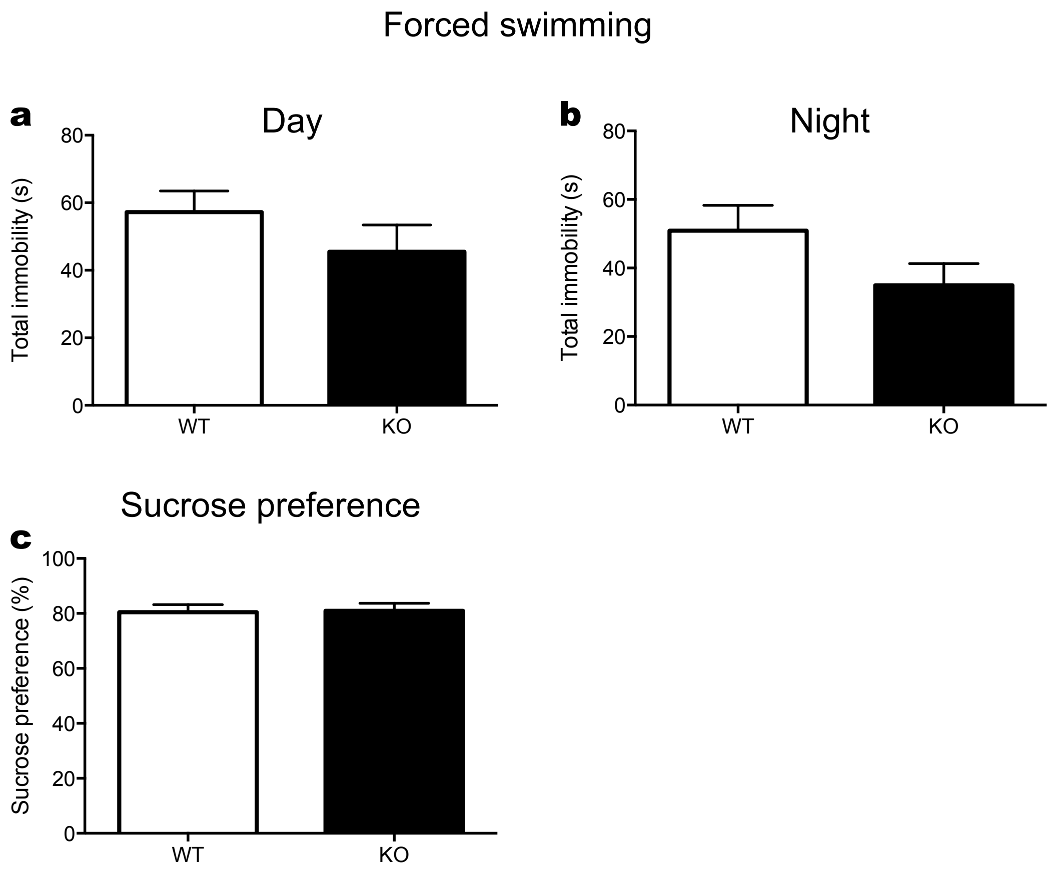

Supplementary Fig. 7: Forced swimming and sucrose preference tests.
No significant differences in forced swimming test in day time (n = 8) (**a**) or night time (n = 10) (**b**). **c,** No difference in sucrose preference (night time) between WT and KO mice (n = 8).

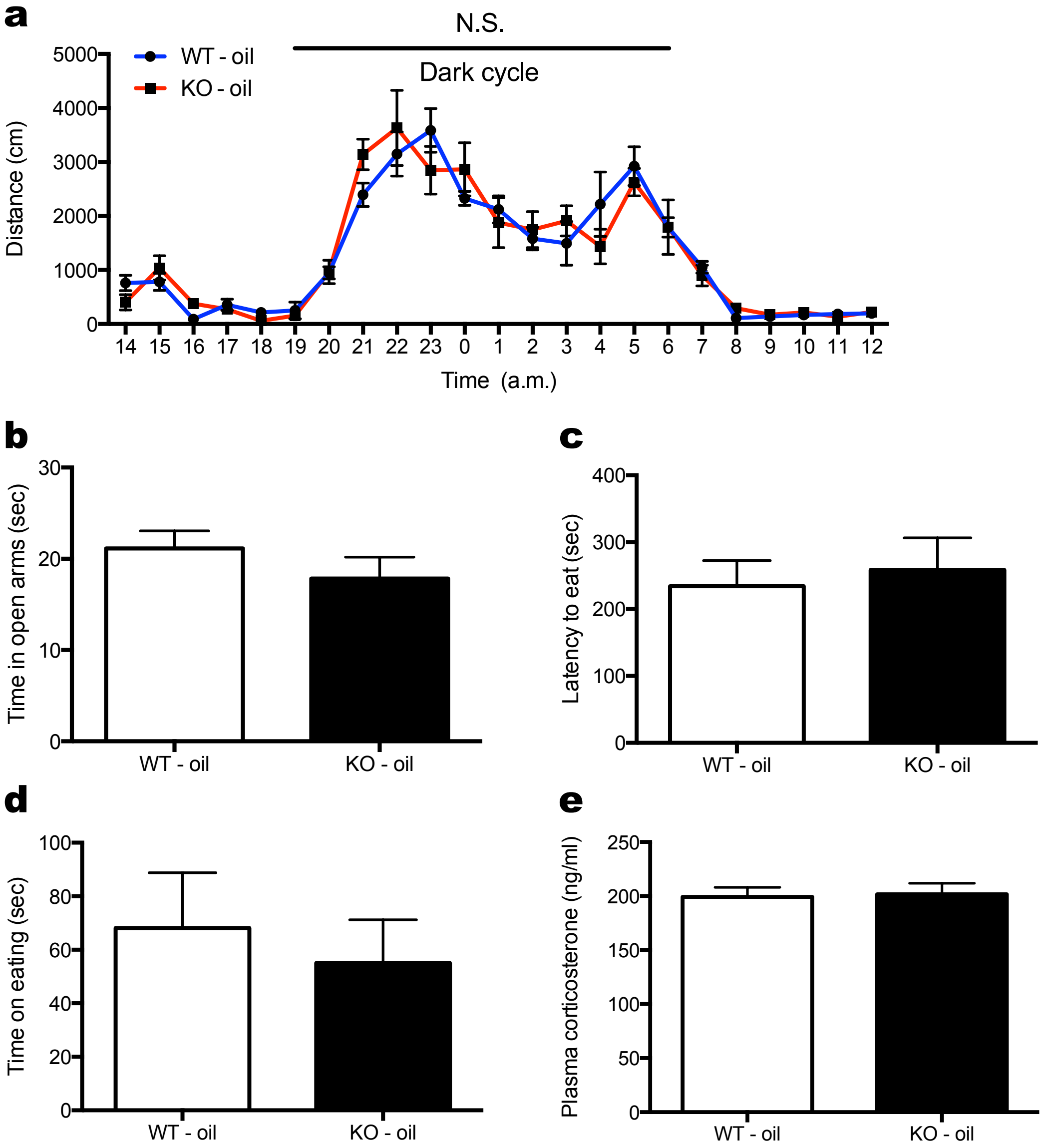

Supplementary Fig. 8: Locomotor activity and anxiety test in oil-injected control. **a,** There was no difference in activity between WT and KO mice with oil injection (n = 8). **b-e,** There was no differences in EPM test (**b, c**) and NSF test (**d, e**) between *Tbr2* inducible KO mice and WT mice with oil injection (n = 8).

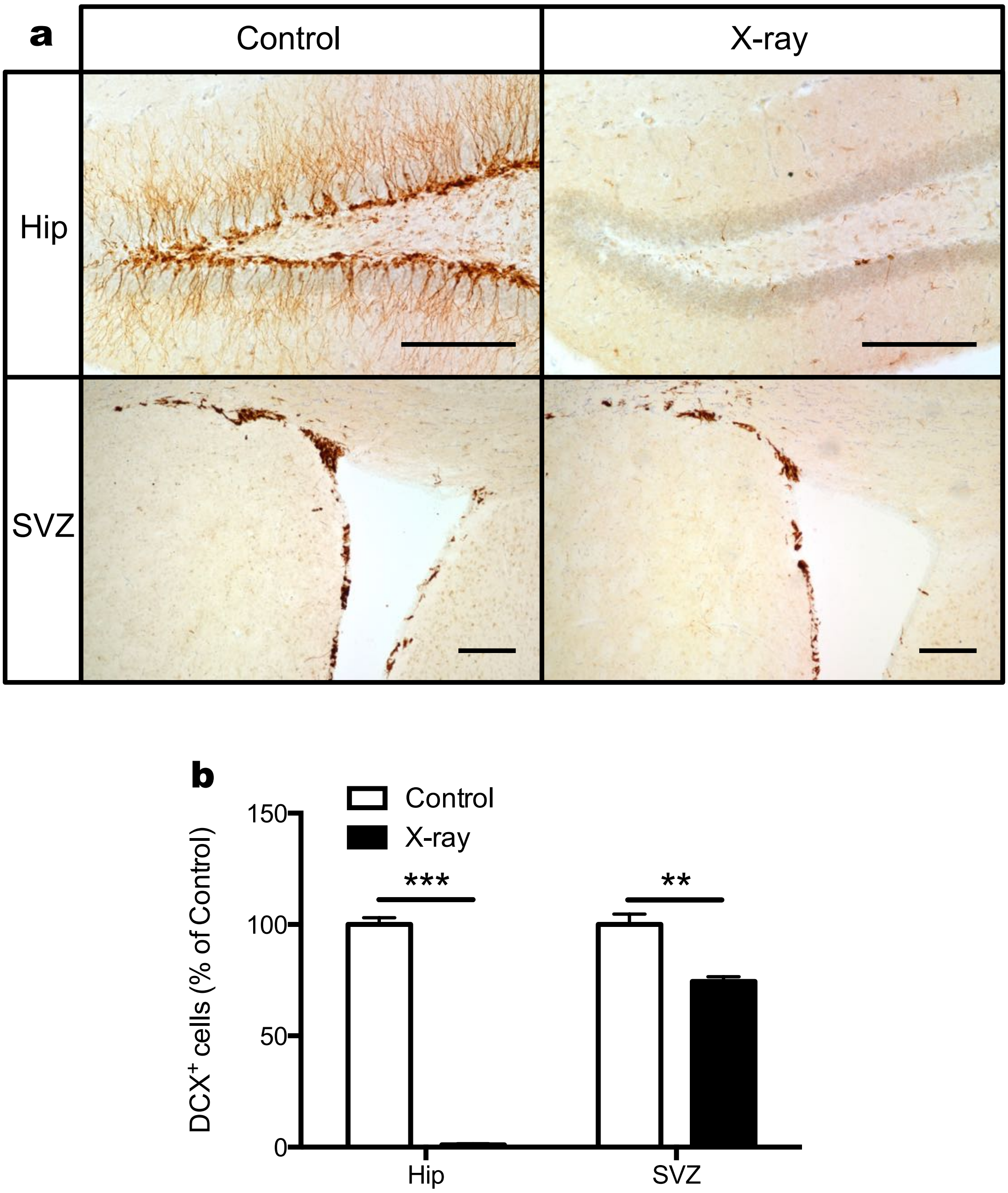

Supplementary Fig. 9: Depleting neurogenesis with X-irradiation.

a, b, Three weeks after X-Irradiation exposure to the middle part of the brain resulted in almost complete depletion of neurogenesis in the dentate gyrus. In contrast 75% DCX^+ cells remain in the SVZ (n = 5). Scale bars = 200 μm.

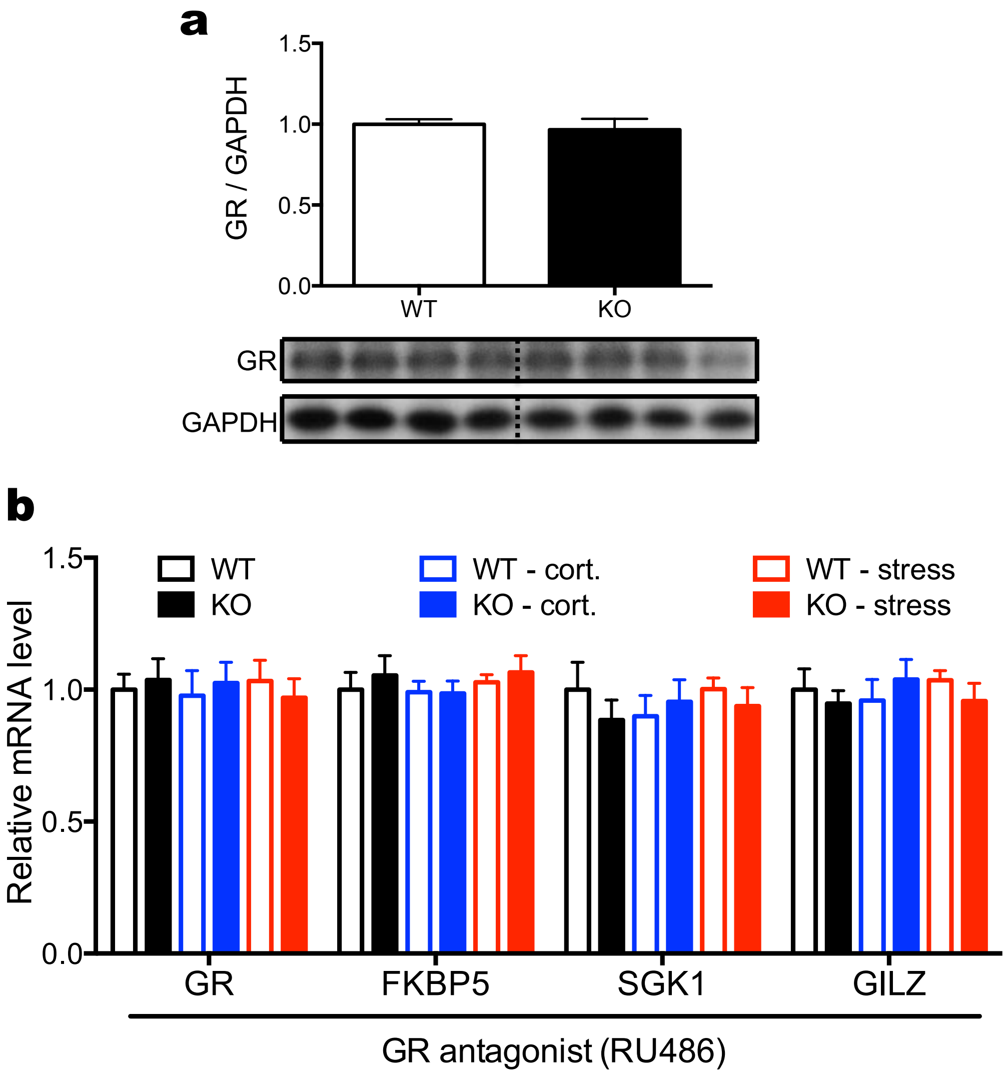

Supplementary Fig. 10: GR protein level in the dentate gyrus and GR antagonist blocked the expression changes in GR target genes.

a, Western blot analysis of GR protein level in the dentate gyrus does not show differences between KO and WT (n = 4). **b,** All mice (n = 6 - 8) were administered RU486 one hour before corticosterone injection or stress: gene expression analysis showed no difference in GR target genes in the hippocampus 30 min after corticosterone injection or stress.

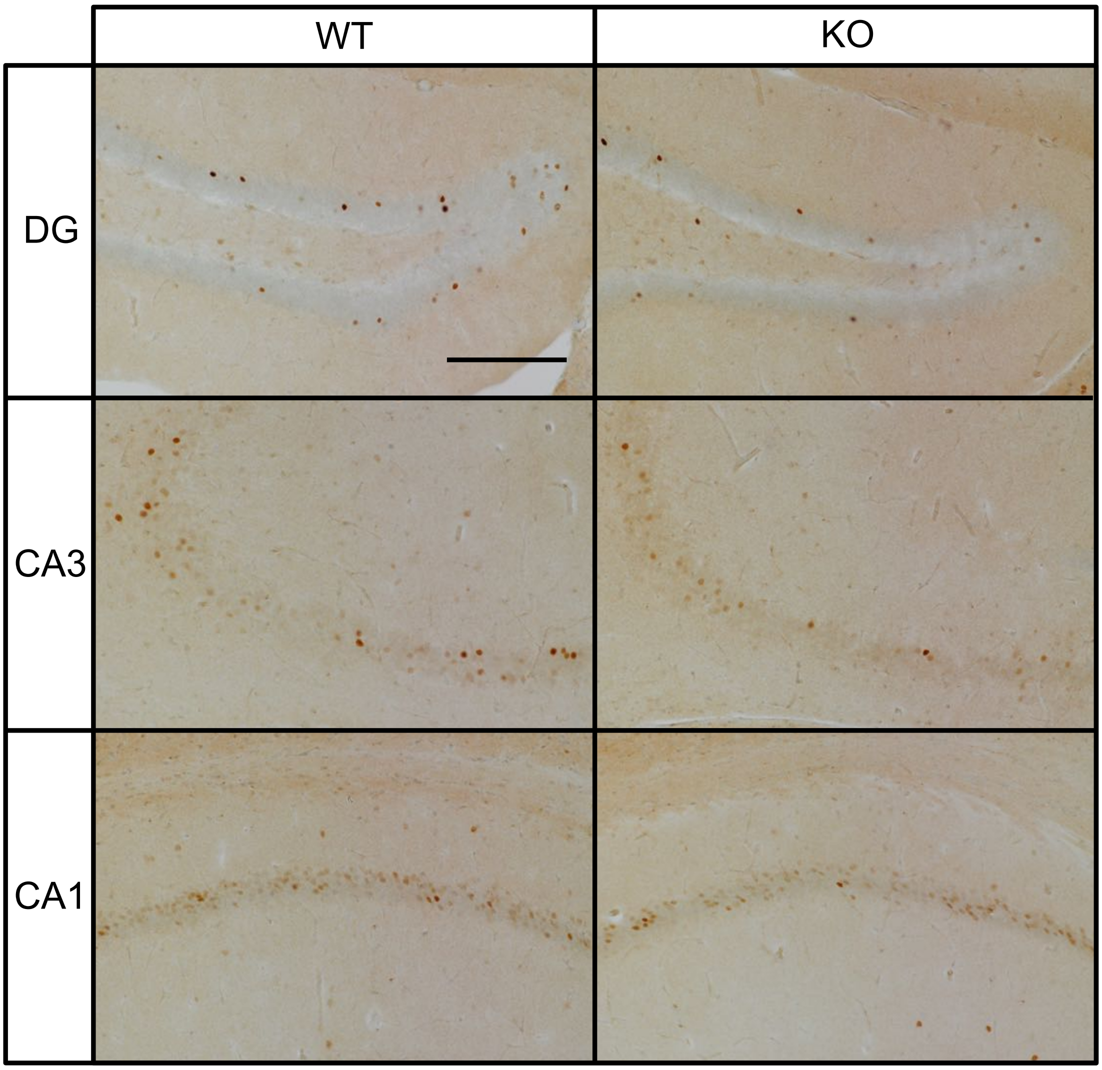

Supplementary Fig. 11: c-Fos$^+$ cells in the hippocampus.

c-Fos immunostaining images showed more c-Fos$^+$ cells in the DG and CA3 of WT mice compared to KO mice, but no difference in CA1 region 90 min after restraint stress started. Scale bars = 200 μm.

Fig. 4a

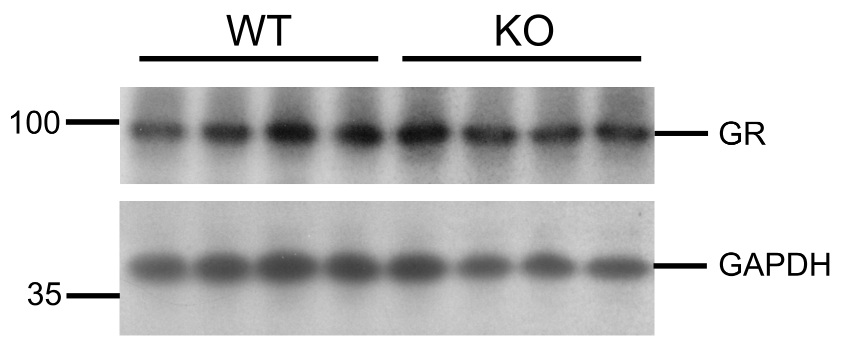

Supplementary Fig. 10a

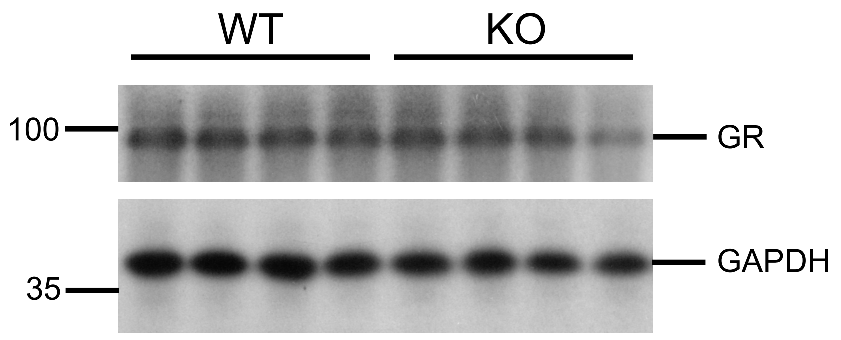

Supplementary Fig. 12: Uncropped scan of western blots.

Uncropped Western blot images of GR and GAPDH (loading control) protein level in the hippocampus (Fig. 4a) and dentate gyrus (Supplementary Fig. 10a).
